# Metallic Glass-Reinforced Metal Matrix Composites: Design, Interfaces and Properties †

**DOI:** 10.3390/ma15238278

**Published:** 2022-11-22

**Authors:** Konstantinos Georgarakis, Dina V. Dudina, Vyacheslav I. Kvashnin

**Affiliations:** 1 School of Aerospace, Transport and Manufacturing, Cranfield University, Cranfield MK43 0AL, UK; 2 Laboratory of Synthesis of Composite Materials, Lavrentyev Institute of Hydrodynamics, Siberian Branch of the Russian Academy of Sciences, Lavrentyev Ave. 15, 630090 Novosibirsk, Russia; 3 Department of Mechanical Engineering, Novosibirsk State Technical University, K. Marx Ave. 20, 630073 Novosibirsk, Russia; 4 Laboratory of Materials Chemistry, Institute of Solid State Chemistry and Mechanochemistry, Siberian Branch of the Russian Academy of Sciences, Kutateladze Str. 18, 630090 Novosibirsk, Russia

**Keywords:** metal matrix composites, metallic glass, amorphous alloy, reinforcement, microstructure, interface, mechanical properties, electrical conductivity

## Abstract

When metals are modified by second-phase particles or fibers, metal matrix composites (MMCs) are formed. In general, for a given metallic matrix, reinforcements differing in their chemical nature and particle size/morphology can be suitable while providing different levels of strengthening. This article focuses on MMCs reinforced with metallic glasses and amorphous alloys, which are considered as alternatives to ceramic reinforcements. Early works on metallic glass (amorphous alloy)-reinforced MMCs were conducted in 1982–2005. In the following years, a large number of composites have been obtained and tested. Metallic glass (amorphous alloy)-reinforced MMCs have been obtained with matrices of Al and its alloys, Mg and its alloys, Ti alloys, W, Cu and its alloys, Ni, and Fe. Research has been extended to new compositions, new design approaches and fabrication methods, the chemical interaction of the metallic glass with the metal matrix, the influence of the reaction products on the properties of the composites, strengthening mechanisms, and the functional properties of the composites. These aspects are covered in the present review. Problems to be tackled in future research on metallic glass (amorphous alloy)-reinforced MMCs are also identified.

## 1. Introduction

In metal matrix composites (MMCs), fibers or particulate inclusions are distributed in a metal matrix. The main purpose of introducing the second phase is mechanical strengthening [1,2,3]. In general, for a given metallic matrix, different reinforcements can be suitable, providing different levels of strengthening. The main design parameters of the composites are (1) the chemical nature (composition) of the matrix and the reinforcement, (2) the concentration, distribution character, and size/morphology of the reinforcement, and (3) the grain size of the matrix. When selecting the reinforcement, its physical and chemical compatibility with the matrix should be analyzed. One of the key concerns is the difference between the coefficients of thermal expansion (CTE) of the metallic matrix and the reinforcement. In the case of ceramic reinforcements, the difference between the CTE of the phases is high, causing a build-up of residual stresses in the composites. Other issues related to the formation of MMCs are the wettability of the reinforcement by the matrix and agglomeration of the reinforcement particles. Poor wettability at the interface causes the formation of pores. When introduced into a metal matrix in the form of agglomerates, particles of high-melting point ceramic materials do not sinter well between each other. This causes the formation of intra-agglomerate pores in MMCs [4].

In order to avoid issues related to the introduction of ceramic particles into metals, soft metals (alloys) can be reinforced by strong metals (alloys) [5,6,7,8,9,10]. Using reinforcements with the same bonding type (metallic bonding) helps avoid the wettability issue. Furthermore, the CTE difference between the phases becomes much smaller. Within this concept, pure (unalloyed) mechanically strong metals can reinforce soft metals. Another possibility is to use intermetallics or multi-component alloys (metallic glasses or high-entropy alloys) to strengthen the soft matrix metals. Reinforcements that sinter well in the same temperature range as the matrix would be even more beneficial for eliminating the intra-agglomerate porosity in the composites. Thus, metallic glasses are very attractive as reinforcements.

Metallic glasses are very interesting materials, and exist in a wide variety of compositions [11,12,13]. At room temperature, metallic glasses demonstrate mechanical behavior different from that of crystalline metals: their elastic strain limit is 2% (the offset strain for crystalline materials is 0.2%) and their strength, hardness, corrosion resistance, and wear resistance usually exceed those of their crystalline counterparts. In the supercooled liquid region ∆T_x_ between the glass transition T_g_ and the crystallization T_x_ temperatures, the mechanical behavior of metallic glasses can be described by Newtonian flow. Metallic glasses deform and sinter easily within the supercooled liquid region ∆T_x_, regaining their strength upon cooling. Although metallic glasses can be designed to possess high strength, they suffer from localized shear and demonstrate little-to-no ductility. However, the ductility lacking in metallic glasses can be provided by a crystalline metal matrix, in which the metallic glass is distributed. Metallic glasses were thought to be able to enter the arena of structural application if combined with ductile metals. This idea was developed in several research groups, who conducted studies of composites containing metallic glass inclusions in a softer phase.

In MMCs, metallic glass inclusions serve as obstacles for the dislocation movement in the matrix or a hard phase partially bearing the load. For the load to be transferred to the metallic glass phase, the latter should be present in high concentrations (in the form of particles, fibers, or a network). Another important condition is strong interface bonding between the glassy reinforcement and the matrix. At the same time, metallic glass contained in the composites acts as a soft binder if the composites are processed within ∆T_x_ [14,15]. It should be kept in mind that the use of metallic glasses as reinforcements for elevated temperature applications is limited due to the tendency of metallic glasses to crystallize above their crystallization temperature T_x_. 

The present review covers composite materials in which the matrix is a crystalline metal or an alloy. Composites with metallic glasses as matrices follow a different pattern of mechanical behavior and are built using a different principle. In those composites, the purpose of the crystalline metal is to reduce the brittleness of single-phase metallic glasses by inducing branching of the shear bands [16,17]. Morphologically, the crystalline phases can be in form of dendrites [18,19], networks [20], 3D printed frames [21], or nanoparticles [22,23,24].

Previous reviews on metallic glass-reinforced MMCs can be found in refs. [25,26,27,28]. The use of metallic reinforcements of amorphous structure in MMCs has also been presented in ref. [5]. As a large number of new reports on structure–property relationships in metallic glass-reinforced MMCs has appeared in the past few years, an update on the state of the art is necessary. 

The present article deals with metallic glass (amorphous alloy)-reinforced MMCs with matrices made of Al, Al alloys, Mg, Mg alloys, Ti alloys, W, Cu, Cu alloys, Ni, and Fe. The early stage of development of this type of composite is also described. 

We dedicate this review to the memory of our teacher and colleague Prof. Alain R. Yavari (1949–2015).

## 2. Early Studies of Metallic Glass (Amorphous Alloy)-Reinforced MMCs

The period between 1982 and 2005 can be regarded as the early stage of development of metallic glass (amorphous alloy)-reinforced MMCs. In this section, works conducted in that period are reviewed [29,30,31,32,33,34,35,36,37] (Table 1). Those works set the stage for the development of this type of composite in the following years.

**Figure 1:**
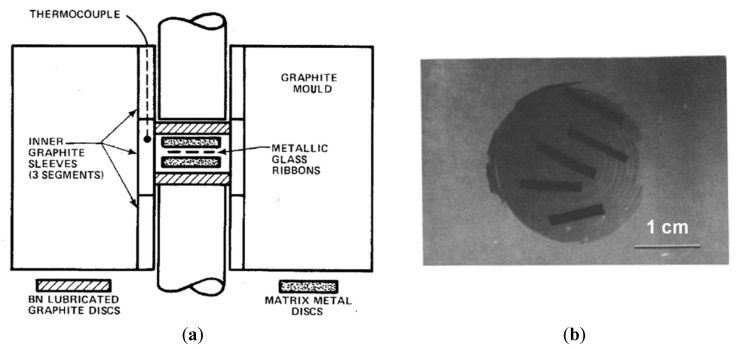
Schematic of the assembly for hot pressing of the ribbons with metal discs (**a**) and a radiograph of the hot-pressed sample (**b**). Reprinted from [29], Copyright (1982), with permission from Springer Nature: Chapman and Hall, Ltd.

In 1982, a study that aimed to demonstrate the possibility of fabricating a composite consisting of an amorphous alloy and a crystalline metal composite was conducted [29]. The reinforcement used was in ribbon form as the least expensive metallic glass product. A wide range of available compositions and a low production cost were put forward as advantages of the amorphous alloy ribbon reinforcements. The ribbons were hot-pressed between discs of an Al alloy 2.3 mm thick (Figure 1). This method of composite fabrication was patented in 1986 [30]. Metallic glass ribbons, wires, and strips were suggested as reinforcements. Those were not dispersed and were used in the as-quenched shapes. The composites were obtained via a solid-state route by placing the metallic glass strips or wires between the Al alloy discs and subjecting the assembly to hot pressing. It was recommended to avoid melting of the metal matrix during the processing of the composites. The temperature of the process was lower than the crystallization temperature of the amorphous alloy.

The first work on composites reinforced with particles of metallic glass appeared in the same year [31]. Composites with a matrix of Al 2014 and particles of a Ni-Mo-Cr-B metallic glass were prepared via the powder metallurgy route, which consisted of mixing, compaction, sintering, and heat treatment. The metallic glass powder and components of the Al-based alloy were mixed and sintered. During liquid-phase sintering, the compacts experienced swelling and the resultant materials remained porous. The sintered compacts were further subjected to re-pressing and re-sintering to reduce porosity. The re-sintered compacts were still rather porous. In the composite sintered from the powder containing 20 vol.% of the metallic glass particles, re-sintering allowed the porosity to decrease from 39% to 33%. The processes occurring in the metallic glass phase during liquid-phase sintering were not studied in detail at that time. The ability of metallic glasses to undergo a glass transition with a concomitant change in viscosity was not yet put forward as an important effect for the processing of composites of this type.

**Figure 2:**
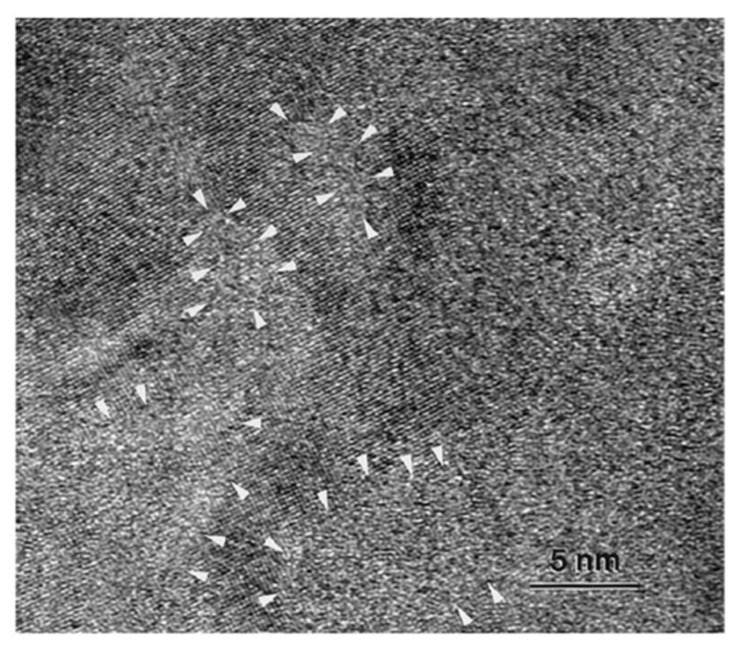
High-resolution transmission electron microscopy image of a rapidly solidified Al-4V-2Fe alloy. Amorphous regions surrounded by the interconnected α-Al phase are arrowed. Reprinted from [38], Copyright (1998), with permission from Elsevier.

In 1995, Inoue et al. [32] reported the formation of Al-V-M (M=Fe, Co, Ni) alloys, in which, upon solidification of the liquid, an amorphous granular phase formed in a matrix of α-Al. The formation of the amorphous particles (in the case of the Al-4V-2Fe alloy, they were about 10 nm in size) was explained by the suppression of the transition of the supercooled liquid to an icosahedral phase. The microstructure of the solidified Al-4V-2Fe material is shown in Figure 2 [38]. The white arrows point to the amorphous regions. The image was taken from the [001] zone of a grain. A fringe contrast corresponding to the [001] zone can be observed. A lack of fringe contrast and a maze-like pattern in certain regions indicates the presence of an amorphous structure. It is seen that some amorphous regions are surrounded by the interconnected α-Al phase. The term “nanoamorphous structure” was used to describe the synthesized alloys. The term “alloy” suits the obtained material better as it was formed via solidification of the liquid. At the same time, since the material has two distinct phases, it can be looked at as a composite structure with unique features. The details of the structure formation process of these alloys can be found in ref. [39].

In 1998, Stawovy & Aning [33] used Fe-40 at.% W alloy particles as a reinforcement for pure iron. The Fe-W powder was obtained via mechanical alloying and contained an amorphous phase and nano-grained tungsten. This powder was blended with a powder of crystalline iron. Bulk Fe + (Fe-W) composites were obtained via cold pressing and annealing.

In 2004, Botta et al. [34] consolidated an Al_90_Fe_7_Zr_3_ powder alloy via severe plastic deformation. The alloy was obtained through mechanical milling and contained crystalline aluminum and an amorphous phase. In the material obtained via high-pressure torsion, the amorphous phase was partially preserved.

**Figure 3:**
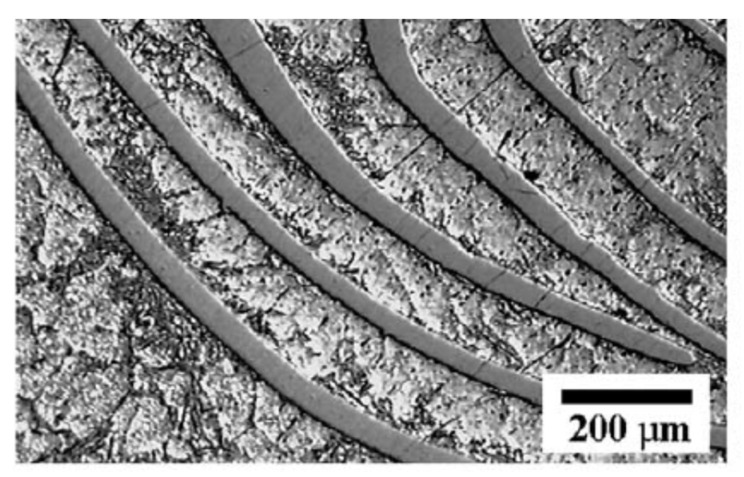
Microstructure of Ni-Nb-Ta metallic glass ribbon-reinforced Al-Si-Mg alloy matrix composite. Reprinted from [35], Copyright (2004), with permission from Elsevier.

The rapid development of metallic glass-reinforced MMCs did not begin until significant progress in the field of metallic glass had been made. The same year, an infiltration-based technology was used for manufacturing a composite of this type. An Al alloy with good castability (Al–6.5 Si–0.25 Mg, wt.%) was chosen as a matrix [35]. The Ni–20.6 Nb–40.2 Ta (wt.%) ribbons were cold-pressed to make a preform for infiltration casting. As the crystallization onset temperature (T_x_) of the amorphous Ni–20.6 Nb–40.2 Ta alloy (699 °C) is higher than the liquidus temperature (T_l_) of the matrix alloy (624 °C), the amorphous phase was preserved in the matrix during the melt infiltration. The microstructure of the obtained metallic glass ribbon-reinforced composite is shown in Figure 3 [35].

**Figure 4:**
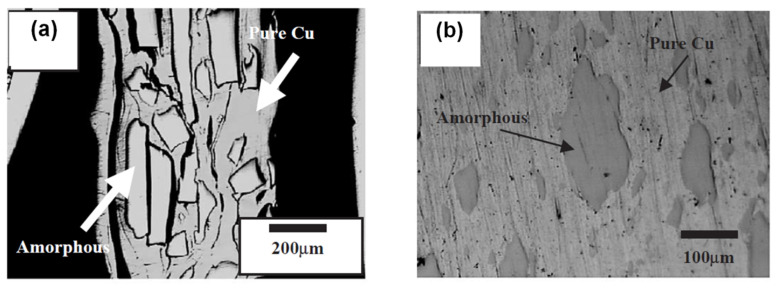
Microstructure of Cu + Ni-Zr-Ti-Si-Sn metallic glass composites obtained via (**a**) cold rolling and folding of Cu foils with Ni-Zr-Ti-Si-Sn metallic glass ribbons, (**b**) warm rolling of cold-rolled composites sealed in a Cu tube. Reprinted from [36], Copyright (2004), with permission from Elsevier.

In 2005, Lee et al. [36] reported the formation of a Ni_59_Zr_20_Ti_16_Si_2_Sn_3_ metallic glass-reinforced Cu matrix composite via a combination of cold rolling, folding, and warm rolling processes. During cold rolling, the metallic glass ribbons broke into shorter pieces while their thickness remained unaffected (Figure 4a). With an increase in the number of rolling and folding cycles, the pieces (particles) of the ribbons became smaller. At the same time, repeated cold rolling caused cracks in the material due to work-hardening of the copper matrix. The warm rolling procedure helped reduce the residual porosity of the material (Figure 4b). The temperature of the warm rolling process was within the supercooled liquid region of the metallic glass. Crystallization of the alloy during warm rolling was prevented. In ref. [36], the usefulness of the viscosity drop within the supercooled liquid region of the metallic glass for the particle reshaping was noted. 

A processing route similar to that described in [33] was used by Wensley et al. [37] to form a Ni matrix composite reinforced with a Ni-W amorphous phase. Two-stage mechanical milling was used to prepare the Ni + (Ni-W) composite powder. At the first stage, partially amorphous Ni-W reinforcement was produced. At the second stage, it was milled with an additional amount of crystalline Ni to produce the target composition. The bulk materials were made via hot-isostatic pressing of the Ni + (Ni-W) composite powder. Upon consolidation, the amorphous phase experienced crystallization.

In the following years, investigations of metallic glass (amorphous alloy)-reinforced MMCs were carried out in a systematic manner and included detailed characterization of the microstructure and mechanical property evaluation. Metallic glasses and matrices of different compositions were used to form composites, as shown in Section 3. The processing conditions were varied to study the evolution of the metallic glass/crystalline metal structures, the interaction of the phases at elevated temperatures, and the formation of reaction products at the interface.

## 3. Approaches to Design and Methods of Fabrication of Metallic Glass (Amorphous Alloy)-Reinforced MMCs

In this section, we review the approaches to the formation of amorphous alloy/crystalline metal structures and methods used for the experimental fabrication of composites. First, an overview of the matrix materials used in the design of metallic glass (amorphous alloy)-reinforced MMCs should be carried out. In Table 2, composites reported to date are classified based on the main element of the matrix. It is seen that, for making the composites, Al, Al alloys, Mg, Mg alloys, Ti alloys, W, Cu, Cu alloys, Ni, and Fe have been used. To the best of our knowledge, 50 papers have been published on the structure and properties of metallic glass (amorphous alloy)-reinforced MMCs with Al or Al alloy matrices. These matrices are attractive from the viewpoint of their application, their relative ease of processing, and the availability and cost of raw materials. It is noteworthy that composites with Cu or Cu-based alloy matrices have been produced recently (2017–2022). 

**Figure 5:**
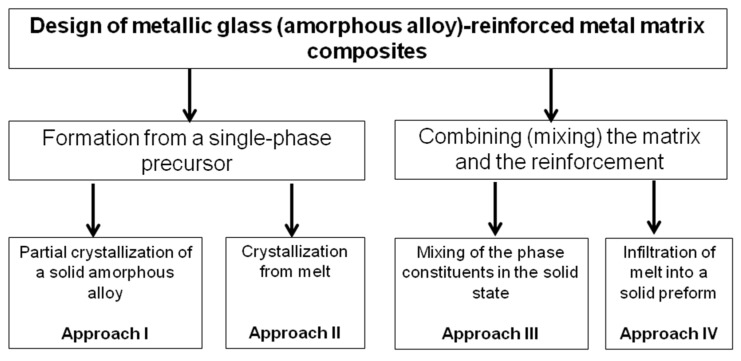
Approaches to the design of metallic glass (amorphous alloy)-reinforced MMCs.

A metallic glass-reinforced MMC is essentially a two-phase (or multi-phase) composite structure in which one phase is amorphous, while the other phases, including the matrix, are crystalline. Approaches to the formation of metallic glass (amorphous alloy)-reinforced MMCs are outlined in the scheme shown in Figure 5. In approaches I and II, the processing starts from a single-phase precursor, which can be either a liquid or an amorphous solid. In approaches III and IV, the amorphous and crystalline phases are combined together (mixed). Experimentally, different methods can be used when following a certain approach (Table 3). The choice of the fabrication method of a composite is dictated by the physical, chemical and mechanical properties of the matrix.

Partial crystallization of amorphous alloy powders leading to the formation of composite structures was studied in refs. [46,63,77]. Sahu et al. [77] showed that composites consisting of α-Al, intermetallics and an amorphous phase can be obtained via spark plasma sintering (SPS) of Al-based alloy powders fabricated through mechanical milling of melt-spun ribbons. In a similar manner, composites containing face-centered cubic (fcc) Al, intermetallics, and an amorphous phase were obtained via SPS of an amorphous powder obtained through mechanical alloying [63].

An amorphous phase can form in situ in a metal matrix when an alloy solidifies from the melt [18,19,32,38]. Alloys with a high content of a crystalline phase can be obtained such that the crystalline phase can be considered a matrix. Hofmann et al. reported the fabrication of composites containing 20 vol.% and 31 vol.% of the glassy phase in the alloys of the Ti-Zr-V-Cu-Al-Be system [19] and 33 vol.% [18] of the glassy phase in the Zr-Ti-Nb-Cu-Be. 

The most versatile approach to the formation of metallic glass (amorphous alloy)-reinforced MMCs is to combine the phases and subsequently consolidate the mixture (assembly), which is the essence of approach III. Particles of both phases can be mixed and consolidated via hot pressing, hot extrusion, microwave sintering, SPS, severe plastic deformation, or rolling (references to articles reporting the use of these methods can be found in Table 3). Since mixing the powders of the matrix and the reinforcement phases and consolidation of the mixtures do not pose any limitations on the composition of the alloys, a variety of composites can be obtained in this manner. The consolidation of the mixtures can be carried out in the supercooled liquid region of the metallic glass or below T_g_ of the glass. For mixtures containing an amorphous alloy without a distinct glass transition, the consolidation is conducted below the crystallization onset temperature of the alloy. In some cases, complete crystallization of the glassy alloy is allowed to occur during consolidation (Table 3).

**Figure 6:**
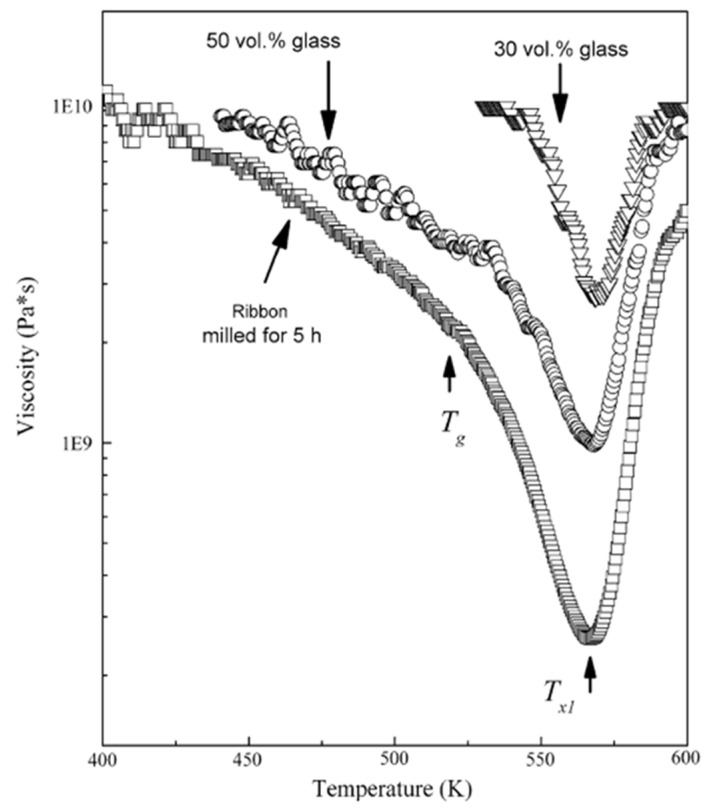
Temperature dependence (heating rate 10 K min^−1^) of the viscosity of the supercooled liquid for the single-phase Al_85_Y_8_Ni_5_Co_2_ glassy ribbon (mechanically milled) and for the Al matrix composites with 50 vol.% and 30 vol.% glass reinforcement. Reprinted from [45], Copyright (2008), with permission from Springer Nature.

Consolidation within the supercooled liquid region is implemented to benefit from the lowered viscosity of the glass, as can be seen in the example described below. Scudino et al. [45] measured the viscosity of the Al_85_Y_8_Ni_5_Co_2_ glassy powder and sintered Al + Al_85_Y_8_Ni_5_Co_2_ composites as a function of temperature using parallel-plate rheometry. A proper consolidation temperature was selected based on the viscosity change of the glassy phase with temperature. As seen in Figure 6, the viscosity dropped when the glass was heated up to 520 K. This viscosity drop was attributed to the structural relaxation. The glass transition occurred above 520 K and was accompanied by a more significant viscosity drop. A temperature of 520 K was selected for consolidation of the composite powders. The viscosity of the consolidated composites also experienced a drop, which confirmed the preservation of the glassy phase in the consolidated state.

**Figure 7:**
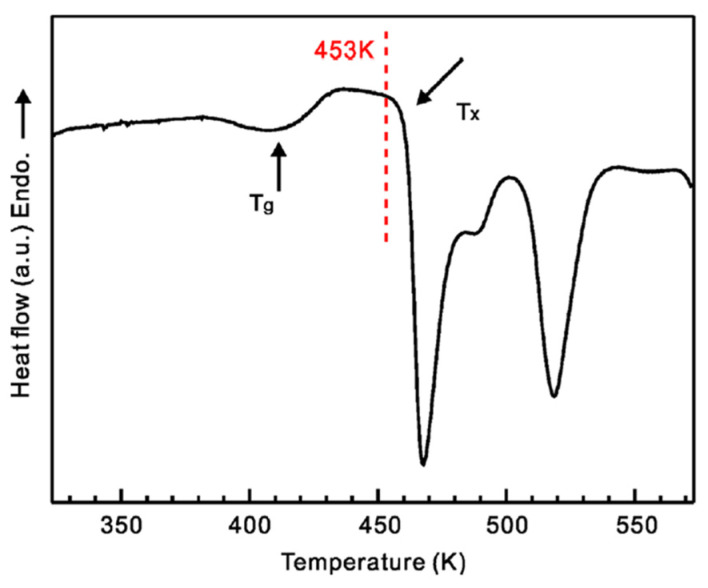
Differential scanning calorimetry scan for the Mg_65_Cu_20_Zn_5_Y_10_ metallic glass. Reprinted from [58], Copyright (2014), with permission from Elsevier.

**Figure 8:**
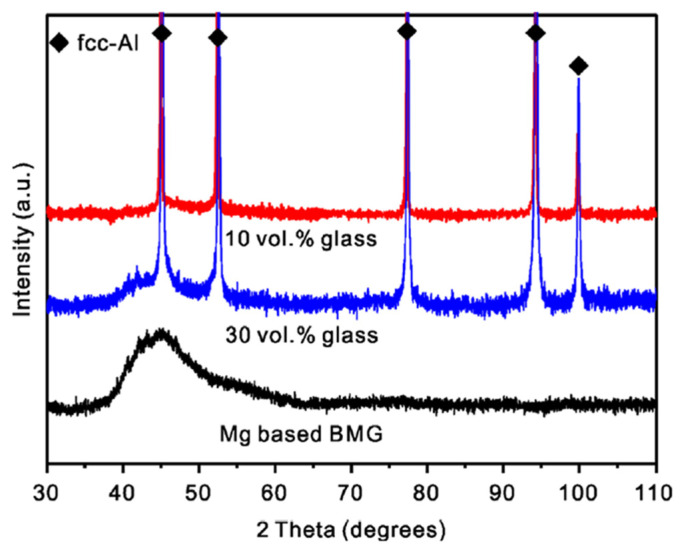
X-ray diffraction (XRD) patterns of the Mg_65_Cu_20_Zn_5_Y_10_ glass and Al + Mg_65_Cu_20_Zn_5_Y_10_ composites. Reprinted from [58], Copyright (2014), with permission from Elsevier.

A challenging, yet attractive, task is to use Al-based or Mg-based metallic glasses for reinforcing aluminum such that lightweight composites can be formed (combining a lightweight matrix with lightweight reinforcement). This task was successfully fulfilled, as reported in refs. [42,58]. For consolidating mixtures of Al with a Mg_65_Cu_20_Zn_5_Y_10_ metallic glass, Wang et al. [58] used high-pressure hot pressing within the supercooled liquid region of the glass (at a temperature of 453 K, Figure 7). The selected consolidation procedure allowed retention of the amorphous structure of the glass, as confirmed by the presence of a halo on the X-ray diffraction pattern of the composites (Figure 8).

**Figure 9:**
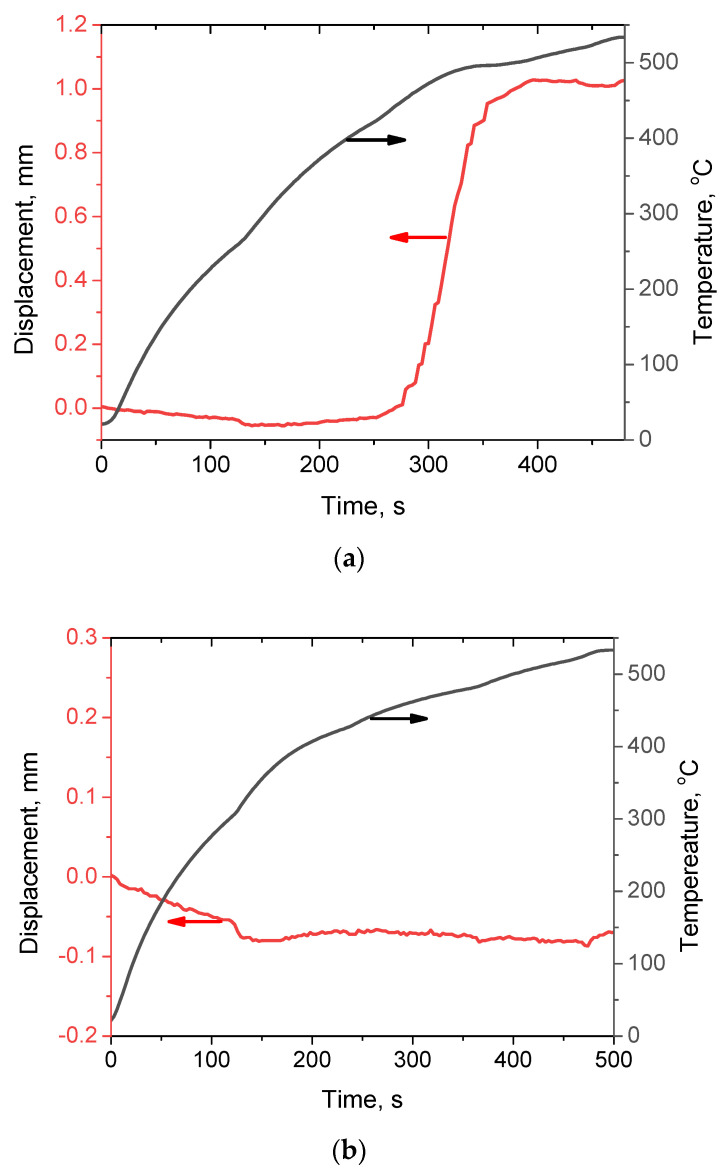
Temperature and displacement versus time during spark plasma sintering (SPS) of the glassy Fe_66_Cr_10_Nb_5_B_19_ (**a**) and crystalline Fe_62_Cr_10_Nb_12_B_16_ (**b**) alloy powders. Black lines—temperature; red lines—displacement. Reprinted from [82]. This article is an open access article distributed under the terms and conditions of the Creative Commons Attribution (CC BY) license (https://creativecommons.org/licenses/by/4.0/).

**Figure 10:**
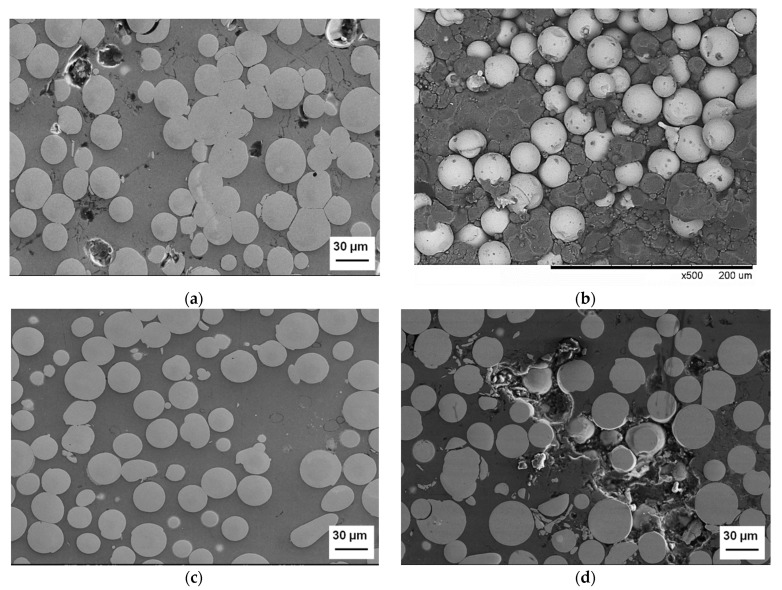
Microstructure of the spark plasma sintered composites obtained from (**a**) Al (coarse)–glassy Fe_66_Cr_10_Nb_5_B_19_, (**b**) Al (coarse)–crystalline Fe_62_Cr_10_Nb_12_B_16_ mixtures, (**c**) Al (fine)–glassy Fe_66_Cr_10_Nb_5_B_19_, (**d**) Al (fine)–crystalline Fe_62_Cr_10_Nb_12_B_16_ mixtures. (**a**,**c**,**d**) Micrographs of the polished cross-sections, SE images; (**b**) micrograph of the fracture surface, BSE image. Reprinted from [82]. This article is an open access article distributed under the terms and conditions of the Creative Commons Attribution (CC BY) license (https://creativecommons.org/licenses/by/4.0/).

**Figure 11:**
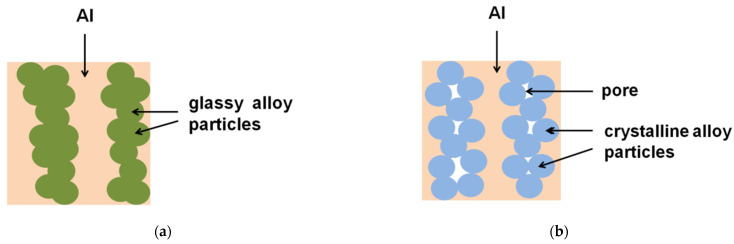

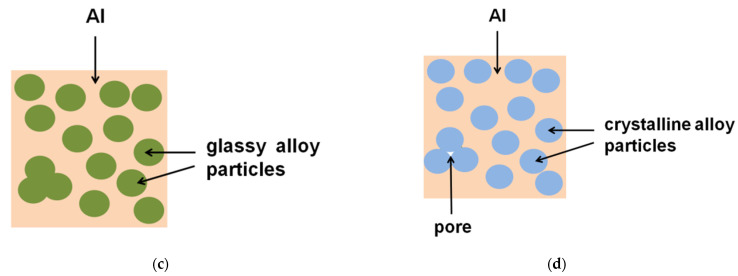
Schematic of the microstructures of aluminum matrix composites formed using a glassy (**a**,**c**) or a crystalline (**b**,**d**) alloy reinforcement. Reprinted from [82]. This article is an open access article distributed under the terms and conditions of the Creative Commons Attribution (CC BY) license (https://creativecommons.org/licenses/by/4.0/).

In [82], the benefit of the glassy reinforcing particles over crystalline particles with a close chemical composition for the densification of Al matrix composites during spark plasma sintering (SPS) was demonstrated. When the Fe-based alloy powders (glassy and crystalline) were heated under pressure, the punch displacement caused by compact shrinkage was observed (Figure 9a) in the case of the glassy alloy when the temperature of the sample reached the glass transition temperature of the alloy (521 °C). The crystalline alloy did not show a tendency to consolidate at this temperature (Figure 9b). Mixtures of an Al + 50 vol.% Fe-based alloy, in which the Fe-based alloy was either a Fe_66_Cr_10_Nb_5_B_19_ metallic glass powder or a crystalline Fe_62_Cr_10_Nb_12_B_16_ alloy, were subjected to SPS via heating up to 540 °C. This temperature is in the supercooled liquid region of the metallic glass (T_g_ = 521 °C, T_x_ = 573 °C). The glassy state of the Fe-based alloy was beneficial for densification, as seen by comparing the images in Figure 10a,c with those shown in Figure 10b,d [82]. The metallic glass played the role of a binder; the densification enhancement effect was more pronounced when the Fe-based alloy particles formed chains (Figure 11) [82]. 

In some cases, the glass transition temperature of metallic glass is difficult to determine from results of differential scanning calorimetry (DSC). Many alloys of amorphous nature do not show a distinct glass transition. Those alloys are still suitable for the role of reinforcement in MMCs. Prashanth et al. [52] proposed a solution for such situations, which is to use the isothermal DSC curves to find the minimum time required before crystallization at a particular temperature. 

Consolidation of the mixtures below the glass transition temperature of the alloy is also possible but may require the use of high pressures (Table 3). Specific conditions are realized in explosive compaction [49], which uses shock waves to compress the powder and form solid bodies under high temperatures (developing locally) and high pressures, acting within a very short period. During compaction, the particle surface can melt. The cooling rates of the melt are in the 10^5^–10^7^ K s^−1^ range, and favor the formation of amorphous alloys in certain compositions. The volume fraction of the material that experienced melting was seemingly insufficient to achieve good bonding between the particles of the mixture, with the consolidated composites failing to reach full density and remaining brittle. 

Metallic glass (amorphous alloy)-reinforced MMCs have been formed using casting technologies (approach IV). Casting a matrix alloy into a preform made of amorphous reinforcing elements can only be applied when the crystallization temperature of the metallic glass T_x_ is higher than the liquidus of the matrix alloy [35,66]. This requirement significantly narrows the choice of materials to be combined in a composite. Furthermore, the alloy should possess good castability, in which case, it can form composites without macrodefects or pores. Another issue pertaining to the processing of metallic glass-reinforced MMCs through casting is a lack of flexibility in varying the reinforcement content. The latter has to be high enough to make a porous preform. Another possibility is to cast the alloy to form the reinforcement phase into a porous skeleton of the metal matrix. It was shown that metallic glass–tungsten composites can be fabricated by making a porous tungsten preform and infiltrating it with a multi-component melt, which transforms upon cooling into a metallic glass [92,94]. This method appears attractive for making composites with refractory metal matrices.

## 4. Interaction of the Amorphous Reinforcement with the Metal Matrix and Evolution of Interfaces

The thermal stability of the metallic glass phase contained in a MMC should be considered not only from the standpoint of the formation of the crystalline products of the same overall composition as that of the initial metallic glass. At processing temperatures of the composites, chemical interactions between the crystalline and amorphous phases may take place at the interfaces. As metallic glasses are multi-component alloys, the situation may be quite complex: the reactivity and diffusivity of several elements will influence the resultant interfacial structure. 

In this context, the compositional effects were studied by Fujii et al. [51], who compared the reactivity of iron and a Fe-based metallic glass towards aluminum under the conditions of friction stir processing. It was found that, under the same heat input, iron was more prone to interaction with aluminum than the Fe_72_B_14.4_Si_9.6_Nb_4_ alloy. The reaction products were observed as layers surrounding the initial iron and metallic glass particles. Additionally, fine particles of Al_13_Fe_4_ were found in the Al matrix. The authors attributed the formation of these particles to the precipitation phenomenon. A higher heat input (a lower travel speed of the tool at a constant rotation speed) was necessary to induce the interaction of Al with the embedded particles in the case of Fe_72_B_14.4_Si_9.6_Nb_4_ than in the case of unalloyed iron.

At temperatures within the supercooled liquid region of the metallic glass, diffusion of the matrix components into the supercooled liquid can occur, with the change in composition causing a reduction in the glass-forming ability of the alloy. This effect was discussed in ref. [89]. Cu-Fe_64_B_24_Y_4_Nb_6_Al_0.4_ metallic glass composites were obtained via friction stir processing, which was performed on copper plates placed on both faces of the metallic glass ribbon. During friction stir processing, the metallic glass ribbon fractured into fragments (particles). Some fragments crystallized and some remained amorphous. In the amorphous particles, clusters of copper were found using transmission electron microscopy. The diffusion of copper into particles of the alloy suggested that the latter reached temperatures above the glass transition of the metallic glass (T_g_ (Fe_64_B_24_Y_4_Nb_6_Al_0.4_) = 858 K). It is also possible that the glass will crystallize first and the crystalline products will react with the matrix. Results obtained by Yu et al. [43] suggest that a significant interaction between the amorphous reinforcement and the metal matrix, resulting in the formation of intermetallic products, takes place after the crystallization of the metallic glass is completed. Lee et al. [35] made a similar observation: a reaction between amorphous ribbons and an Al alloy occurred only after the glass had crystallized. 

Depending on the processing conditions of the composites, the thickness of layers with a structure different from those of the starting phases can vary, ranging from several nanometers [65] to several micrometers [80,81]. Owing to the complex chemistry of the amorphous reinforcement, the products of its interaction with the matrix are usually composed of several intermetallic phases. The interfacial bonding strength is a key feature of a composite. Strong bonding at the interface enables efficient load transfer from the matrix to the reinforcement. If the reinforcing particles are poorly bonded to the matrix, the overall strengthening becomes inefficient. The formation of thick (of the order of several micrometers) intermetallic layers between the matrix and the reinforcement particles can further strengthen the composites, as the growth of the layer means that the volume fraction of phases harder than the matrix increases. Rapid sintering techniques using the application of electromagnetic fields are efficient in preventing devitrification of the reinforcement and controlling the interfacial reactions [28]. At the same time, Guan et al. [75] suggest that, during SPS, surface crystallization of metallic glass particles can be induced. Under a passing electric current, local overheating at the inter-particle contact areas increases the growth rate of the reaction product layers between the matrix and the metallic glass [80].

**Figure 12:**
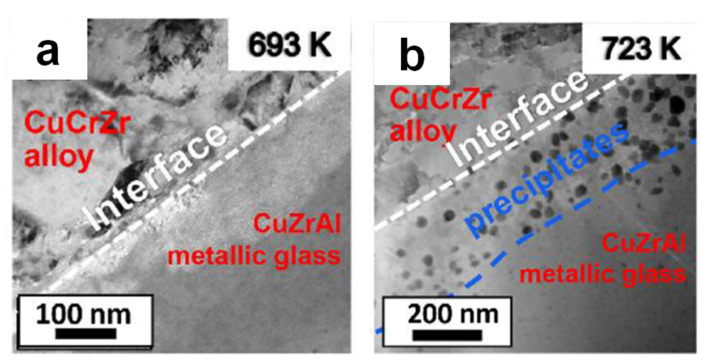
Transmission electron microscopy images of the interface in composites obtained via spark plasma sintering of CuCrZr alloy + 30 wt.% Cu_50_Zr_43_Al_7_ metallic glass mixtures at 500 MPa at different temperatures: (**a**) 693 K and (**b**) 723 K. Reprinted from [88], Copyright (2022), with permission from Elsevier.

Figure 12 shows transmission electron microscopy images of the interface in composites obtained via SPS of CuCrZr alloy + 30 wt.% Cu_50_Zr_43_Al_7_ metallic glass powder mixtures at a pressure of 500 MPa and temperatures of 693 K and 723 K [88]. A tight bond between the reinforcement and the matrix in the composite sintered at 420 °C is seen in Figure 12a. The metallic glass does not experience crystallization during SPS at this temperature. At 723 K, nanocrystals precipitate at the interfacial region (Figure 12b) owing to crystallization of the CuZrAl metallic glass. This is an important observation showing that the CuZrAl metallic glass crystallizes first at the interface. Two reasons were suggested for the observed structural change: initial high energy of the interface and diffusion at the interfacial region. 

**Figure 13:**
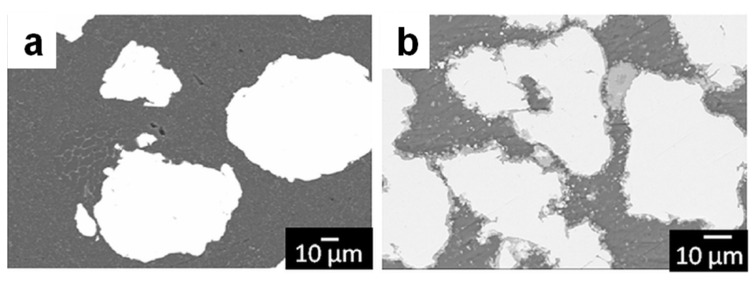
Microstructure of composites obtained from Al 2024 + 40 vol.% Ni_60_Nb_40_ mixtures: (**a**) as-hot-pressed state and (**b**) heat-treated state. Reprinted from [71], Copyright (2019), with permission from Elsevier.

The changes in the microstructure and phase composition due to the interfacial reactions in Al 2024 + 40 vol.% Ni_60_Nb_40_ composites resulted in a significant strength enhancement [71]. Figure 13a shows a micrograph of the hot-pressed material, in which no reaction has occurred. Evidence of the interfacial reactions between the matrix and the reinforcement is seen in the hot-pressed composites subjected to heat treatment (Figure 13b). A layer of the reaction products surrounds the Ni-Nb particles in the heat-treated composites. This layer contains mainly Al, Cu, and Ni. The source of Cu and Al is the matrix alloy, while that of Ni is the reinforcing particles. It was noted that the formation of these layers enabled more efficient load transfer from the matrix to the particles.

**Figure 14:**
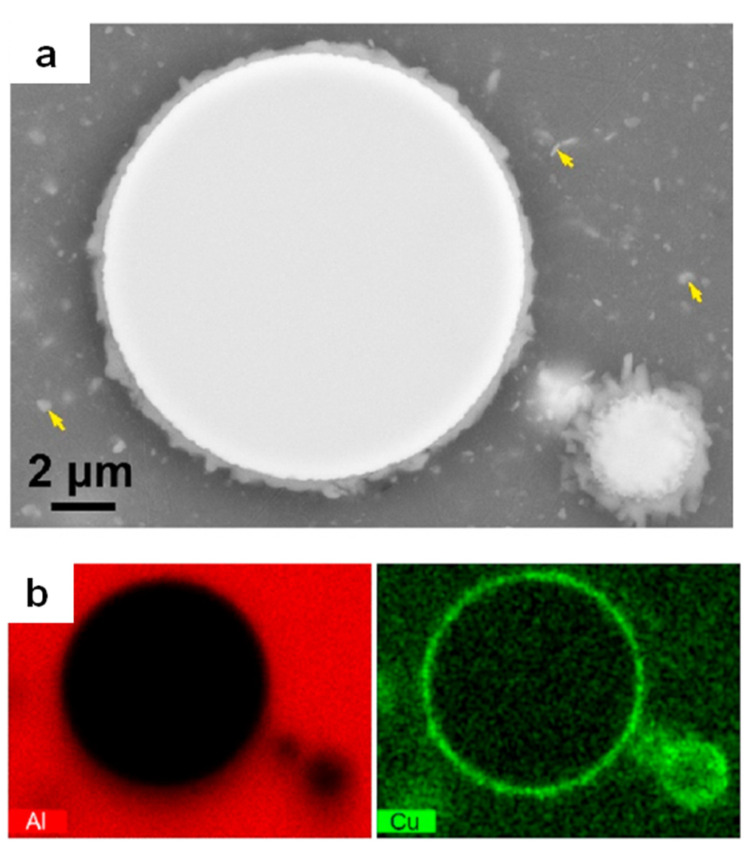
(**a**) A micrograph showing a particle of the Fe_43.2_Co_28.8_B_19.2_Si_4.8_Nb_4_ metallic glass in the matrix of Al 2024 alloy. The composite was heat-treated. (**b**) Maps of Al and Cu; the map of Cu shows its concentration at the interface. Reprinted from [78], Copyright (2020), with permission from Elsevier.

An interesting situation developed in the composites in ref. [78]: the interaction of the added particles with the matrix led to matrix depletion of the alloying element. In that work, Al 2024 matrix composites with distributed Fe-based glass particles were heat-treated to study the interaction of the metallic glass inclusions with the matrix and its effect on the mechanical properties of the composites. The Al_7_Cu_2_Fe intermetallic phase formed at the interface (Figure 14). The thickness of the intermetallic layer increased with increasing treatment temperature and time. The formation of the intermetallic phase reduced the concentration of Cu in the matrix alloy, ultimately weakening precipitation hardening of the matrix. Additionally, the formation of the intermetallic layer lowered the bonding strength between the particles and the matrix, making particle debonding upon failure of the composites possible. Overall, the chemical interaction between the added metallic glass and the matrix led to a reduction in the strength of the composites. For example, the heat-treated (480 °C, 25 min), quenched, and naturally aged composite obtained from the Al 2024 + 20 vol.% Fe_43.2_Co_28.8_B_19.2_Si_4.8_Nb_4_ showed a tensile yield strength of 317 MPa, an ultimate tensile strength of 438 MPa, and a fracture strain of 4.8%, while the unreinforced alloy subjected to the same treatment was stronger, demonstrating a tensile yield strength of 390 MPa, an ultimate tensile strength of 631 MPa, and a fracture strain of 19.1%. This study showed that the chemical composition of the matrix and metallic glass reinforcement should be carefully selected for composites, which are processed under conditions favoring the interaction of the matrix and the reinforcement. 

Amorphous-crystalline laminate materials of unique structure were reported by Wu et al. [95]. The crystal–glass symbiotic alloys were formed via magnetron sputtering through alternate deposition of 18 nm thick Cr-Co-Ni nanolayers and 12 nm thick Ti-Zr-Nb-Hf nanolayers. The mutual elemental partitioning among the adjacent phases modified their individual properties (the effect was called symbiotic). Dynamic partitioning of Ni and Co from the crystalline Cr-Co-Ni phase to the amorphous Ti-Zr-Nb-Hf-Cr-Co-Ni phase occurred. The negative mixing enthalpy of the glass was enhanced; at the same time, the crystalline alloy became more ductile due to partial phase transformation.

While the above-discussed examples are concerned with systems differing in their scale and architecture, they show the possibility of mutual influence of the phases in crystalline–amorphous composites. The interfaces between a metallic glass and a crystalline metal (alloy) are crucial not only in particle- or ribbon-reinforced composites but also in welds [96,97,98,99] and sintered layered structures [50]. In a broader context, metallic glass–crystalline metal interactions are special cases of interactions between multi-component alloys and metals, which play a key role in the structure formation of metallic alloy particle-reinforced composites [100,101]. Therefore, studies of the chemical interaction and diffusion between metallic materials of different crystalline structure and composition are of fundamental importance for many systems.

## 5. Properties of Metallic Glass (Amorphous Alloy)-Reinforced MMCs

### 5.1. Mechanical Properties

The following strengthening mechanisms usually operate in metallic glass (amorphous alloy) particle-reinforced composites: load transfer from the matrix to the reinforcing phase, grain boundary strengthening (Hall–Petch effect), Orowan strengthening, and dislocation strengthening [54,65,87,88]. As for Orowan strengthening, for which fine particles are required, it can also operate in the matrix alloy itself in the case of alloys containing precipitate phases. Solution strengthening is another contribution to the total strengthening, if the matrix of the composite is a solid solution [87,88].

The structural design of composites aimed at strength enhancement can be realized by reducing the size of the reinforcing particles and/or the grain size of the matrix. Several examples of the influence of the structural refinement on the mechanical behavior of metallic glass-particle-reinforced composites are discussed below. 

Mechanical milling is an efficient method to mix the powders and refine the structure of the matrix [62,70] or the composite as a whole [15,54,67,87]. In ref. [62], the Al powder was subjected to preliminary milling before mixing with the powder of the Cu_43_Zr_43_Al_7_Ag_7_ metallic glass. The use of a nanostructured matrix was shown to significantly increase the strength of the Al + Cu_43_Zr_43_Al_7_Ag_7_ composites. 

In the study presented in [70], mechanical milling of the powders of the Al matrix and metallic glass did not result in a decrease in the metallic glass particle size. However, the composite that was hot-pressed from a mixture milled for 10 h was stronger than that obtained from the mixture milled for 1 h only. This behavior was attributed to an accumulation of defects in the matrix itself during milling. 

**Figure 15:**
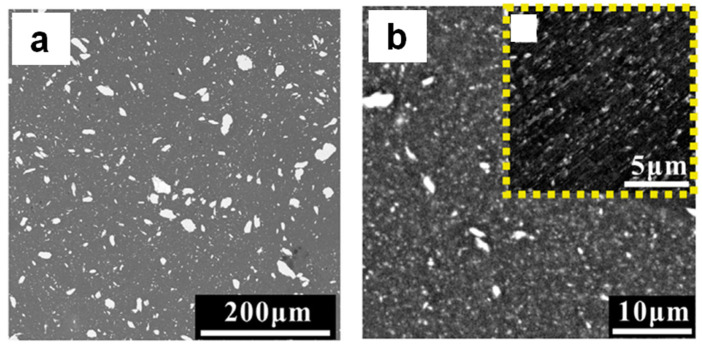
Microstructure of the hot-extruded composites obtained from Al 7075 + 8 vol.% Ti_52_Cu_20_Ni_17_Al_11_ mixtures milled for 10 h (**a**) and 50 h (**b**). Reprinted from [67], Copyright (2018), with permission from Elsevier.

**Figure 16:**
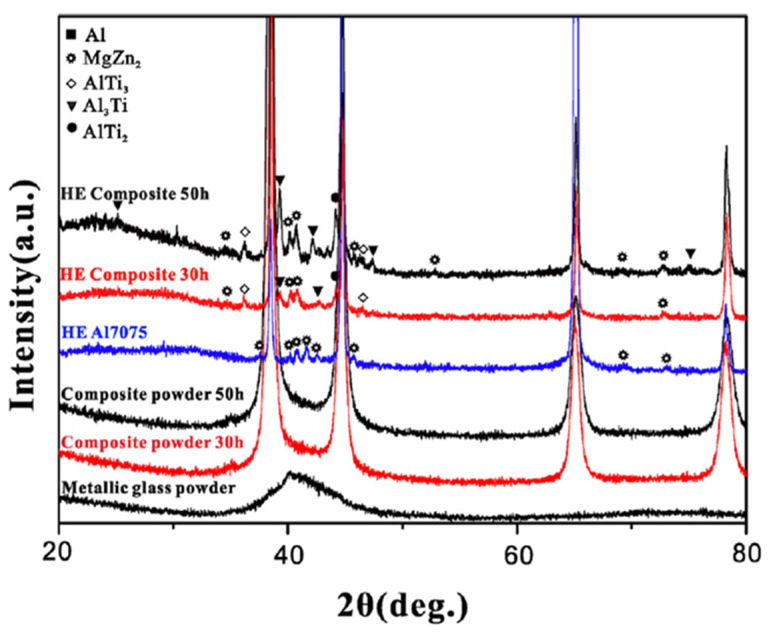
XRD patterns of the Ti_52_Cu_20_Ni_17_Al_11_ metallic glass_,_ Al 7075 + 8 vol.% Ti_52_Cu_20_Ni_17_Al_11_ mixtures, hot-extruded matrix alloy, and composites obtained from the Al 7075 + 8 vol.% Ti_52_Cu_20_Ni_17_Al_11_ mixtures milled for 30 h and 50 h. Reprinted from [67], Copyright (2018), with permission from Elsevier.

The effect of the milling time on the mechanical properties of the hot-extruded composites obtained from the Al 7075 + 8 vol.% Ti_52_Cu_20_Ni_17_Al_11_ mixture was studied in [67]. The powder mixture was milled for 10–50 h. After 50 h of milling, the size of the reinforcing particles was in the nanometer range (Figure 15). The formation of AlTi_3_, Al_3_Ti, and AlTi_2_ in the hot-extruded composite indicates partial crystallization of the glassy reinforcement (Figure 16). The authors consider grain refinement, Orowan strengthening, and dislocation–dislocation interactions as the most significant contributors to the enhanced strength of the hot-pressed nanocomposite. It was also noted that micrometer-sized particles were still present in the composites formed from mixtures milled for 50 h; those particles reduced the fracture strain of the composites. 

Another example of the influence of the milling time of the powder mixtures on the mechanical properties of the composites can be found in ref. [87] for the Cu-Cr-Zr + 30 wt.% Cu-Zr-Al system. As the milling time increased, the metallic glass particles became finer and transformed into strips with widths between 5 μm and 15 μm. Both the yield strength and the ultimate compressive strength of the composites increased with the milling time of the mixture. Thus, the yield strength of the composite sintered from the mixture milled for 30 h was 1365 MPa, while that of the composite sintered from the non-milled sample was only 645 MPa. The ultimate compressive strength values of the composites were 926 MPa and 1433 MPa for 0 h and 30 h of milling, respectively. The contributions of different strengthening mechanisms (grain boundary, metallic glass particle, solid solution and dislocation strengthening) to the yield strength of the composites were assessed. The high yield strength of the composites was attributed mainly to grain boundary strengthening and metallic glass particle strengthening. These contributions increased with the milling time of the powder mixture, which can be explained by the grain refinement of the matrix and metallic glass particle reduction upon milling.

Table 4 presents the compressive property data of selected metallic glass (amorphous alloy)-reinforced composites extracted from the literature. The composites are grouped based on the main element of the matrix material. An analysis of the mechanical property data of the composites together with their microstructural data allowed us to draw the following conclusions:

(1) The use of metallic glass as reinforcement for MMCs enables materials with high strength and ductility to be obtained; an attractive combination of properties has also been achieved in composites with fully or partially crystallized reinforcements.
(2) To achieve high strengthening levels, it is necessary to use fine particles of metallic glass; powders obtained via gas atomization (and not subjected to subsequent milling for size reduction) do not produce significant strengthening effects when introduced into matrices.
(3) The matrix of the composite should be fine-grained, which is ensured by using certain procedures, such as mechanical milling and non-equilibrium (fast) consolidation of the powders.
(4) The matrix/reinforcement interactions affect the mechanical properties of the composites; the “sign” of this effect depends on the chemical properties of the constituents.

**Figure 17:**
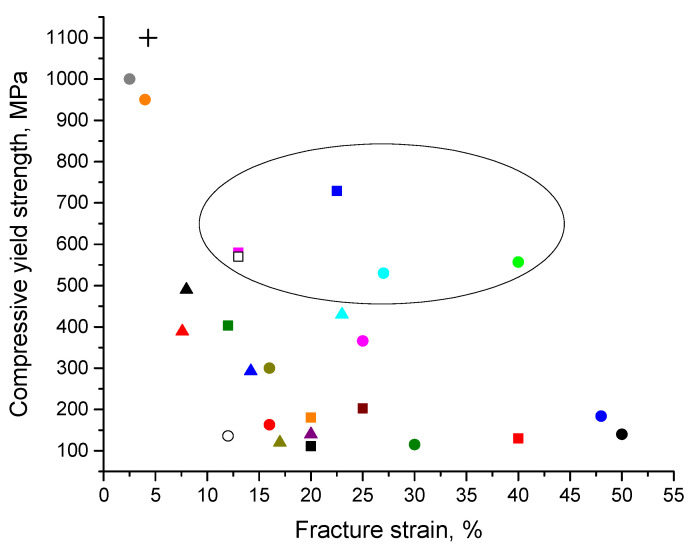
Compressive yield strength–fracture strain data for Al and Al alloy matrix metallic glass (amorphous alloy)-reinforced composites: ■ Al + Ni_70_Nb_30_ [40]; ■ Al + Al_85_Y_8_Ni_5_Co_2_ [45]; ■ Al 5083 + Al_85_Ni_10_La_5_ [41]; ■ Al 520.0 + Cu_54_Zr_36_Ti_10_ [15]; ■ Al + Mg_58_Cu_28.5_Gd_11_Ag_2.5_ [60]; ■ Al + Mg_65_Cu_20_Zn_5_Y_10_ [58]; ■ Al 2024 + Fe_73_Nb_5_Ge_2_P_10_C_6_B_4_ [59]; ● Al + Fe_66_Cr_10_Nb_5_B_19_ [81]; ● Al-Si-Mg + Ni–Nb–Ta [35]; ● Al + Al-Cu-Ti [64]; ● Al 7075 + Zr_65_Cu_18_Ni_7_Al_10_ [65]; ● Al 7075 + Ti_48_Zr_7.5_Cu_39_Fe_2.5_Sn_2_Si_1_ [74]; □ Al 6061 + [(Fe_1/2_Co_1/2_)_75_B_20_Si_5_]_96_Nb_4_ [54]; ● Al 7075 + Ti_55.5_Cu_18.5_Ni_17.5_Al_8.5_ [76]; ○ Al + Fe_50.1_Co_35.1_Nb_7.7_B_4.3_Si_2.8_ [70]; ● AlSi10Mg + Ni_60_Nb_20_Ta_20_ [66]; ✚ Al + Al_65_Cu_16.5_Ti_18.5_ [68]; ●, ● Al 7075 + Ti_52_Cu_20_Ni_17_Al_11_ [67]; ▲, ▲ Al 2024 + Ni_60_Nb_40_ [71]; ▲, ▲, ▲ Al + Cu_43_Zr_43_Al_7_Ag_7_ [62]; ▲ Al + Zr_48_Cu_36_Ag_8_Al_8_ [69]; ● Al + Fe_74_Mo_4_P_10_C_7.5_B_2.5_Si_2_ [61].

In Figure 17, the compressive yield strength and fracture strains of composites with Al and Al alloy matrices are plotted for a more convenient analysis. An oval marks composites demonstrating relatively a high yield strength and a large fracture strain. These composites are based on Al alloys as matrices. It was interesting to answer the question of whether the same combinations of properties can be achieved in ceramic particle-reinforced composites with the same matrices. Table 5 is presented to compare the compressive yield strength and fracture strain values of composites with metallic glass reinforcements (or their derivatives) and ceramic reinforcements. As seen in Table 5, Al 5083 was reinforced by boron carbide, B_4_C [102,103]. The compressive yield strength and fracture strain of the composite reported in [103] were lower than the corresponding values of the composite reinforced by Al_85_Ni_10_La_5_ [41]. As for the composite obtained in [102], it had the advantage of higher strength but at the cost of limited plasticity. The Al 6061 alloy reinforced with MgAl_2_O_4_ ceramic particles [104] showed a lower compressive yield strength and a lower fracture strain than the composite with the same matrix reinforced with a Fe-Co-based metallic glass [54]. B_4_C-reinforced Al 7075 [105] showed only a slightly higher compressive yield strength (600 MPa) than composites with the same matrix reinforced with Ti-based metallic glasses (530 MPa [67]; 557 MPa [76]). At the same time, the plasticity of the composites reinforced with metallic glass was much better than that of the B_4_C-containing composite (27% and >40% for the metallic glass-reinforced composites versus 20% for the ceramic particle-reinforced composite).

**Figure 18:**
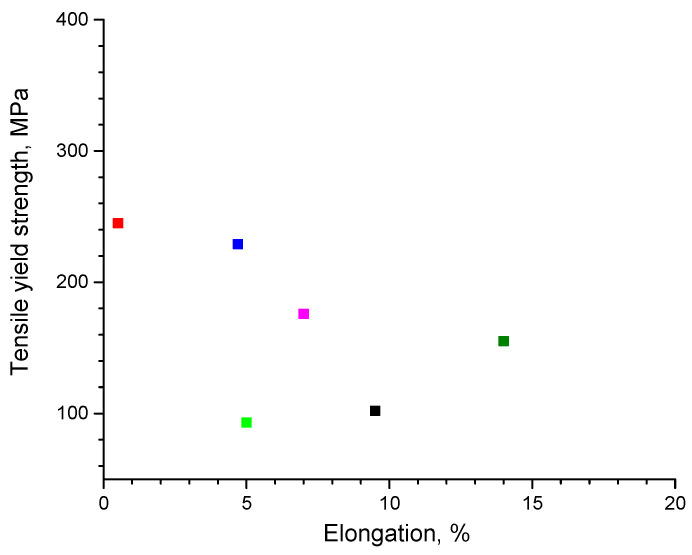
Tensile yield strength–elongation data for Al and Al alloy matrix composites: ■ Al + Ni_60_Nb_40_ [55]; ■ Al-Zn-Ca + Co_48_Cr_15_Mo_14_C_15_B_6_Tm_2_ [73]; ■, ■ Al 2024 + Fe_49.9_Co_35.1_Nb_7.7_B_4.5_Si_2.8_ [57]; ■ Al + Al_84_Gd_6_Ni_7_Co_3_ [56]; ■ Al + Zr_48_Cu_36_Ag_8_Al_8_ [69].

The tensile property data for metallic glass (amorphous alloy)-reinforced MMCs are still rather limited. Table 6 presents the tensile properties of selected composites with different matrices. In Figure 18, the data for composites with Al and Al alloy matrices are plotted. It is seen that certain progress has been made in the composite design: alloys with reasonably high elongations have been obtained. The next step should be to increase the strengthening levels to those reached under compressive loading. It is worth noting the attractive properties of Ti-based and Zr-Ti matrix composites obtained via semisolid processing [18,19].

### 5.2. Wear Resistance

The metallic glass-reinforced Al matrix composites showed remarkably improved wear resistance relative to that of the unreinforced matrix when subjected to abrasive pin-on-disc tests [52]. The abrasive wear rate decreased from 2.46 × 10^−4^ m^3^/m for the Al 6061 extruded alloy to 0.373 × 10^−4^ m^3^/m for the composite containing 50 vol.% of the glass. Plowing was found to be the dominant mechanism of wear of the unreinforced alloy. The addition of a glassy reinforcement with higher hardness than the matrix material reduced the interaction between the hard abrasive particles and the soft matrix, which resulted in diminished groove formation, as indicated by lower surface roughness.

### 5.3. Electrical Conductivity

**Figure 19:**
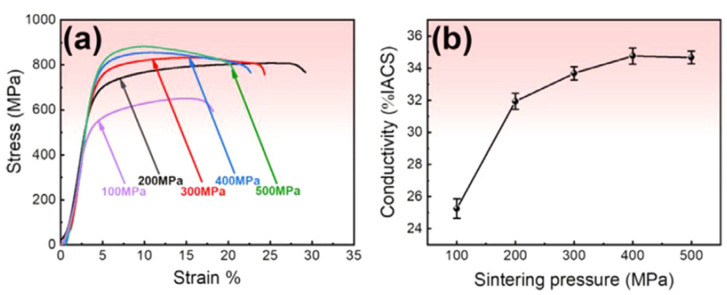
(**a**) Engineering stress–strain curves of composites obtained via SPS of CuCrZr alloy + 24 wt.% Cu_50_Zr_43_Al_7_ metallic glass mixtures at 693 K and different pressures; (**b**) variation in the electrical conductivity of the composites with the sintering pressure. Reprinted from [88], Copyright (2022), with permission from Elsevier.

The electrical conductivity of MMCs is an important characteristic which is challenging to maintain at a high level when significant mechanical strengthening is required. For composites with enhanced strength and electrical conductivity, copper is the best candidate for the matrix. Recent publications report the electrical conductivity of metallic glass-reinforced Cu alloy matrix composites [86,87,88]. In ref. [88], the mechanical properties and electrical conductivity of composites obtained via SPS of CuCrZr alloy + 24 wt.% Cu_50_Zr_43_Al_7_ mixtures at 693 K and different pressures are presented. The strength (Figure 19a) and electrical conductivity (Figure 19b) of the composites increased with increasing sintering pressure. The property improvement was attributed to the enhanced interfacial bonding in composites sintered at higher pressures. In the sintered CuCrZr alloy + 30 wt.% Cu_50_Zr_43_Al_7_ composites (450 °C, 500 MPa), a combination of strength exceeding 850 MPa and electrical conductivity exceeding 32% of the International Annealed Copper standard was achieved.

### 5.4. Corrosion Resistance of Metallic Glass-Reinforced MMCs

Reports on the corrosion resistance of metallic glass (amorphous alloy)-reinforced MMCs are still scarce [31,72]. Generally, metallic glasses demonstrate much higher corrosion resistance than crystalline metals. However, it should be kept in mind that the corrosion rate of the matrix can increase in the presence of metallic glass inclusions compared with an unreinforced matrix metal. So, the overall corrosion behavior of the composites should be evaluated experimentally. Jha et al. [31] tested the corrosion resistance of Al 2014 reinforced with particles of a Ni-Mo-Cr-B metallic glass. It was found that the corrosion rate in an artificial sea-water environment decreased linearly with the volume fraction of the metallic glass dispersoids. It was also shown that, in order to decrease the corrosion rate, the residual porosity of the sintered composites should be reduced. 

Zhou et al. [72] recorded the polarization curves of composites sintered from Al + Fe_52_Cr_15_Mo_26_C_3_B_1_Y_3_ metallic glass powder mixtures. A 3.5% NaCl solution was used for the tests. When compared with aluminum, the composites obtained from mixtures containing 5–20 vol.% of the glass demonstrated better corrosion resistance (more positive corrosion potentials and lower corrosion current densities). The corrosion current of the composite sintered from the Al + 15 vol.% Fe_52_Cr_15_Mo_26_C_3_B_1_Y_3_ was found to be two orders of magnitude lower than that of unreinforced aluminum.

## 6. Future Research Directions

Although significant advancements in the area of metallic glass (amorphous)-reinforced MMCs have been made in recent years, there is still a lot of room for improvement in the design of these composites at the microstructural level. Novel composites can be formed by changing the elemental composition of the matrix and reinforcement, the size of the metallic glass inclusions, and the degree of interaction of the metallic glass with the matrix.

Comparative studies of the mechanical properties of composites reinforced by particles of a metallic glass and crystalline particles of the same elemental composition, with all other microstructural parameters (residual porosity, particle size, and particle distribution uniformity in the matrix) equal, would shed light on the role of the crystalline structure of the reinforcement in the strength enhancement of the composite. Reinforcements with a partially crystallized structure are also of interest. For some compositions, at the early stages of crystallization of the glass, an increase in the hardness of the material occurs due to the precipitation of compounds with high hardness from an amorphous matrix [106]. 

In future investigations, it may be interesting to introduce metallic glass fibers or ribbons in the metal matrix in an ordered pattern. The continuous reinforcing elements can be aligned or placed in a certain 3D geometry to design composites with new sets of properties.

Most studies conducted so far on metallic glass (amorphous alloy)-reinforced composites have focused on the mechanical behavior of these materials. The functional properties of these composites are yet to be tested and optimized. For metallic glass-reinforced Cu matrix composites, their electrical conductivity values and dependence on processing parameters have recently become available. Investigations in the area of corrosion and wear resistance and the corresponding mechanisms are also required for these types of composites.

A transition from laboratory practice to real-life applications is still to be made. For this, the structural evolution and damage accumulation in these composites need to be evaluated in operando mode. An important consideration is the cost of the constituents of the metallic glasses to be used as reinforcements. Therefore, a compromise between the properties of the glass and its cost should be reached.

## 7. Summary

At present, metallic glasses and amorphous alloys are considered to be promising alternative reinforcements for metals and alloys. The main advantages of metallic alloys of an amorphous nature are high mechanical strength and the presence of bonds of a metallic type, which makes them compatible with metallic matrices. Until now, in research laboratories, metallic glass (amorphous alloy)-reinforced composites with matrices made of Al, Al alloys, Mg, Mg alloys, Ti alloys, W, Cu, Cu alloys, Ni, and Fe have been produced. Different approaches to the formation of metallic glass (amorphous alloy)-reinforced MMCs can be used. The processing can start from a single-phase precursor, which is either a liquid or an amorphous solid. The amorphous and crystalline phases can be combined (mixed) to produce a composite structure via solid-state consolidation or infiltration. The consolidation of powder mixtures is the most versatile approach to manufacturing metallic glass (amorphous alloy)-reinforced MMCs. If the consolidation of ribbons or powders of metallic glass mixed with metallic powders is conducted within the supercooled liquid region of the glass, the latter acts as a binder, helping densification. 

The thickness of the interfacial layers formed between the metallic glass (amorphous alloy) and the matrix depends on the processing conditions and mutual chemistry of the phases. The product of interaction can be a thin interdiffusion layer (several nanometers thick) or a thick layer composed of crystalline phases. The influence of the formation of thick layers of the reaction products on the mechanical properties of the composites depends on the chemical composition of the system. In some systems, the presence of the added inclusions in the matrix can alter the composition and mechanical strength of the matrix alloy.

The introduction of metallic glass in Al alloy matrices has enabled composites with increased mechanical strength and large fracture strains to be obtained. Recent reports on Cu alloy matrix composites reinforced with metallic glass particles indicate the possibility of forming composites possessing both high strength and high electrical conductivity. In future studies of this type of composite, it would be interesting to determine their wear and corrosion resistance, as well as the underlying mechanisms of the observed phenomena.

## Figures and Tables

**Table 1:** Early studies on metallic glass (amorphous alloy)-reinforced metal matrix composites (MMCs).

Year	Material Description (Matrix + Reinforcement Added/Formed In Situ)	Reference
1982	Al-Ca-Zn + Ni-Nb amorphous alloy (added)	[29]
1986	Al-Ca-Zn + Ni-Nb amorphous alloy (added)	[30]
1986	Al 2014 + Ni-Mo-Cr-B metallic glass (added)	[31]
1995	Al + (Al-V-Fe) amorphous alloy (formed in situ during solidification)	[32]
1998	Fe + (Fe-W) partially amorphous alloy (added)	[33]
2004	Al + Al-Fe-Zr amorphous alloy (formed in situ during mechanical milling and partially preserved after consolidation)	[34]
2004	Al-Si-Mg + Ni-Nb-Ta metallic glass (added)	[35]
2005	Cu + Ni-Zr-Ti-Si-Sn metallic glass (added)	[36]
2005	Ni + (Ni-W) partially amorphous alloy (added)	[37]

**Table 2:** Classification of metallic glass (amorphous alloy)-reinforced MMCs based on the main element of the matrix.

Matrix Composition	References
Al and its alloys	[15,29,31,32,34,35,38,40,41,42,43,44,45,46,47,48,49,50,51,52,53,54,55,56,57,58,59,60,61,62,63,64,65,66,67,68,69,70,71,72,73,74,75,76,77,78,79,80,81,82]
Mg and its alloys	[14,83,84]
Ti alloys	[18,19]
Cu and its alloys	[85,86,87,88,89,90]
Ni	[37]
Fe	[33]
W	[91,92,93,94]

**Table 3:** Fabrication methods of metallic glass (amorphous alloy)-reinforced MMCs.

Approach	Fabrication Method	References
I	Partial crystallization of amorphous alloy powders during consolidation	[46,63,77]
II	Semisolid processing	[18,19]
II	Melt quenching	[32,38]
III	The reinforcement is metallic glass; consolidation at a temperature within the supercooled liquid region of the metallic glass:	
	- Induction heating sintering (hot pressing)	[14,15,54]
	- Conventional sintering followed by induction heating sintering (hot pressing)	[72]
	- High-pressure * induction heating sintering	[59]
	- Hot pressing followed by hot extrusion	[45,47]
	- High-pressure * hot pressing	[58,60]
	- Spark plasma sintering	[53,82]
	- Equal-channel angular extrusion	[91]
	- Friction stir processing	[48,89]
	- Warm rolling	[36]
III	The reinforcement is metallic glass; consolidation at a temperature below the glass transition of the metallic glass:	
	- Spark plasma sintering	[79]
	- High-pressure * spark plasma sintering	[65,88]
	- High-pressure * hot pressing followed by hot extrusion	[57,78]
III	The reinforcement is an amorphous (or partially amorphous) alloy, which does not show a distinct glass transition; consolidation below the crystallization onset temperature:	
	- Equal-channel angular pressing	[64]
	- Cold pressing followed by conventional sintering	[40,44]
	- Hot pressing	[29,30,44]
	- Cold pressing followed by conventional sintering and hot extrusion	[44]
	- Microwave sintering followed by hot extrusion	[55,83,84]
	- High-pressure consolidation followed by annealing and rolling	[42]
	- Hot isostatic pressing	[37]
	- Hot-roll bonding	[73]
	- Friction stir processing/welding below the glass transition temperature of the metallic glass	[51]
III	Consolidation accompanied by complete devitrification of the added amorphous alloy:	
	- Hot extrusion	[41,56]
	- Spark plasma sintering	[90]
III	Rapid consolidation (temperatures are high enough to induce melting):	
	Explosive compaction	[49]
IV	Melt infiltration into a porous preform made of amorphous alloy ribbons or flakes	[35,66]
IV	Infiltration of a porous crystalline metal preform with a melt solidifying into a glassy phase;	[92]
	The same followed by hydrostatic extrusion	[94]

* In the context of this review, high-pressure processes are those involving pressures of several hundred MPa.

**Table 4:** Mechanical properties of selected metallic glass (amorphous alloy) particle-reinforced MMCs in compression.

Composition of the Material (Matrix + Added Reinforcement)	Processing Method and Conditions	Compressive Yield Strength, MPa	Ultimate Compressive Strength, MPa	Strain at Fracture/at Maximum Stress (Marked *), %	Reference
**Al and Al Alloy Matrix Composites**					
Al + 30 wt.%Ni_70_Nb_30_	Conventional sintering, 773 K, 2 h	111	146	>20	[40]
Al + 30 wt.%Ni_60_Nb_40_	Conventional sintering, 823 K, 30 min; hot extrusion	134	-	-	[44]
Al + 30 vol.% Al_85_Y_8_Ni_5_Co_2_	Hot pressing; hot extrusion, 520 K	120	255	>40/10 *	[45]
Al + 50 vol.% Al_85_Y_8_Ni_5_Co_2_	Hot pressing; hot extrusion, 520 K	130	295	>40/7 *	[45]
Al + 40 vol.% Zr_57_Ti_8_Nb_2.5_Cu_13.9_Ni_11.1_Al_7.5_	Hot pressing; hot extrusion	-	200	70	[47]
Al + 60 vol.% Zr_57_Ti_8_Nb_2.5_Cu_13.9_Ni_11.1_Al_7.5_	Hot pressing; hot extrusion	-	250	40	[47]
Al 5083 + 10 vol.% Al_85_Ni_10_La_5_ (fully crystallized during processing)	Hot extrusion; swaging	729	-	22.5	[41]
Al 520.0 + 15 vol.% Cu_54_Zr_36_Ti_10_	Induction heating sintering, 720 K, 50 MPa	580	840	13	[15]
Al + 30 vol.% Mg_58_Cu_28.5_Gd_11_Ag_2.5_	Hot pressing, 453 K, 700 MPa	180	212	>20	[60]
Al + 10 vol.% Mg_65_Cu_20_Zn_5_Y_10_	Hot pressing, 453 K, 700 MPa	203	247	25	[58]
Al 2024 + 15 wt.% Fe_73_Nb_5_Ge_2_P_10_C_6_B_4_	Induction heating sintering, 823 K, 30 min, 400 MPa	403	660	12	[59]
Al + 20 vol.%Fe_66_Cr_10_Nb_5_B_19_	SPS, 843 K, 3 min, 40 MPa	-	780	2	[81]
Al + 20 vol.%Fe_66_Cr_10_Nb_5_B_19_	SPS, 813 K, 0 min, 40 MPa	110	-	>50	[81]
Al + 20 vol.%Fe_66_Cr_10_Nb_5_B_19_	SPS, 813 K, 0 min, 40 MPa; forging	140	-	>50	[81]
Al + 20 vol.%Fe_66_Cr_10_Nb_5_B_19_	SPS, 813 K, 3 min, 40 MPa	130	-	>50	[81]
Al + 15 vol.% Fe_52_Cr_15_Mo_26_C_3_B_1_Y_3_	Conventional sintering, 823 K, 2 h; hot pressing, 793 K, 40 MPa	-	234	1.8	[72]
Al + 30 vol.% Al_70_Y_16_Ni_10_Co_4_	Hot pressing, 673 K; hot extrusion, 673 K	131	-	-	[52]
Al + 50 vol.% Al_70_Y_16_Ni_10_Co_4_	Hot pressing, 673 K; hot extrusion, 673 K	163	-	-	[52]
Al 6061 + 30 vol.% Al_70_Y_16_Ni_10_Co_4_	Hot pressing, 673 K; hot extrusion, 673 K	211	-	-	[52]
Al 6061 + 50 vol.% Al_70_Y_16_Ni_10_Co_4_	Hot pressing, 673 K; hot extrusion, 673 K	238	-	-	[52]
Al-Si-Mg + 20 vol.% Ni–Nb–Ta	Infiltration of the molten matrix alloy into a pressed preform of ribbons	163	320	16	[35]
Al + 10 vol.% Al-Cu-Ti	Equal-channel angular pressing, 523 K	184	-	48	[64]
Al + 40 vol.% Zr_57_Cu_20_Al_10_Ni_8_Ti_5_	SPS, 663 K, 3 min, 100 MPa	-	225	7.5	[53]
Al 7075 + 16 vol.% Zr_65_Cu_18_Ni_7_Al_10_	SPS, 573 K, 10 min, 600 MPa	366	471	25	[65]
Al 7075 + 15 vol.% Ti_48_Zr_7.5_Cu_39_Fe_2.5_Sn_2_Si_1_	SPS, 573 K, 10 min, 600 MPa	950	1002	4	[74]
Al 6061 + 15 vol.% [(Fe_1/2_Co_1/2_)_75_B_20_Si_5_]_96_Nb_4_	Induction heating sintering, 828 K, 2 min, 70 MPa	570	600	13	[54]
Al + 40 vol.% Fe_74_Mo_4_P_10_C_7.5_B_2.5_Si_2_	Hot pressing, 673 K, 600 MPa	115	245	30/24 *	[61]
Al 7075 + 6 vol.% Ti_55.5_Cu_18.5_Ni_17.5_Al_8.5_	Hot extrusion, 673 K	557	618	>40	[76]
Al + 60 vol.% Fe_50.1_Co_35.1_Nb_7.7_B_4.3_Si_2.8_	Milling, 1 h; hot pressing, 673 K, 30 min, 640 MPa	46	225	25	[70]
Al + 60 vol.% Fe_50.1_Co_35.1_Nb_7.7_B_4.3_Si_2.8_	Milling, 10 h; hot pressing, 673 K, 30 min, 640 MPa	136	282	12	[70]
AlSi10Mg-based alloy + 36 vol.% Ni_60_Nb_20_Ta_20_(along flake orientation direction)	Melt infiltration; heat treatment (annealing, 798 K; quenching; artificial aging, 438 K	300	430	16	[66]
Al + 40 vol.% Al_65_Cu_16.5_Ti_18.5_	SPS, 773 K, 400 MPa	1100	1710	4.3	[68]
Al 7075 + 8 vol.% Ti_52_Cu_20_Ni_17_Al_11_	Milling, 10 h; hot extrusion, 673 K	530	570	27	[67]
Al 7075 + 8 vol.% Ti_52_Cu_20_Ni_17_Al_11_	Milling, 50 h; hot extrusion, 673 K	~1000	~1000	2.5	[67]
Al 2024 + 40 vol. Ni_60_Nb_40_	Hot pressing, 673 K, 640 MPa	293	490	14.2	[71]
Al 2024 + 40 vol. Ni_60_Nb_40_	Hot pressing, 673 K, 640 MPa; heat treatment (annealing, 773 K, 1 h; quenching; aging, 423 K, 18 h)	389	620	7.6	[71]
Al (nanostructured) + 40 vol.% Cu_43_Zr_43_Al_7_Ag_7_	Hot pressing, 673 K, 600 MPa, 10 min	490	560	8	[62]
Al (nanostructured) + 20 vol.% Cu_43_Zr_43_Al_7_Ag_7_	Hot pressing, 673 K, 600 MPa, 10 min	430	530	23	[62]
Al + 20 vol.% Cu_43_Zr_43_Al_7_Ag_7_	Hot pressing, 673 K, 600 MPa, 10 min	120	150	17	[62]
Al + 20 vol.% Zr_48_Cu_36_Ag_8_Al_8_	Hot pressing, 673 K, 640 MPa; hot extrusion, 673 K	140	-	>20	[69]
**Mg and Mg alloy matrix composites**					
Mg + 5 vol.% Ni_60_Nb_40_	Microwave sintering; annealing, 673 K, 1 h; hot extrusion, 623 K	130	320	18.4	[83]
Mg + 10 vol.% Ni_50_Ti_50_	Microwave sintering; annealing, 673 K, 1 h; hot extrusion, 673 K	102	417	15	[84]
Mg AZ91 + 15 vol. % Zr_57_Nb_5_Cu_15.4_Ni_12.6_Al_10_	Induction heating sintering, 713 K, 2 min, 50 MPa	325	542	11	[14]
**Cu and Cu alloy matrix composites**					
Cu + 50 wt.% Cu_39.2_Zr_36_Al_4.8_Ni_10_Ti_10_	Hot pressing, 663 K, 1 GPa, 4 h	400	490	8 *	[85]
Cu + 50 wt.% Cu_39.2_Zr_35.2_Al_5.6_Ni_10_Ti_10_	Hot pressing, 663 K, 1 GPa, 4 h	420	470	10 *	[85]
Cu-Cr-Zr + 30 wt.% Cu-Zr-Al	Non-milled, SPS, 693 K, 500 MPa	645	926	25	[87]
Cu-Cr-Zr + 30 wt.% Cu-Zr-Al	Ball milling, 5 h, SPS, 693 K, 500 MPa	900	1100	14	[87]
Cu-Cr-Zr + 30 wt.% Cu-Zr-Al	Ball milling, 30 h, SPS, 693 K, 500 MPa	1365	1433	7.5	[87]
Cu + 40 vol.% Fe-Si-B (in situ devitrified)	SPS, 1073 K, 40 MPa	548	957	29	[90]
**Ni matrix composites**					
Ni + 25 vol.% (Ni-W)	Hot isostatic pressing, 193 MPa, 973 K, 30 min	620	1120	21	[37]
Ni + 45 vol.% (Ni-W)	Hot isostatic pressing, 193 MPa, 973 K, 30 min	1020	1200	17	[37]
**W matrix composites**					
W + 40 vol.% Zr_58.5_Nb_2.8_Cu_15.6_Ni_12.8_Al_10.3_	Equal-channel angular extrusion, 697 K	-	1540	2	[91]
W + 50 vol.% Hf_44.5_Cu_27_Ni_13.5_Ti_5_Al_10_	SPS, 300 MPa	1020	1143	1.1	[93]
W + 30 vol.% Hf_44.5_Cu_27_Ni_13.5_Ti_5_Al_10_	SPS, 300 MPa	832	1115	1.7	[93]

**Table 5:** Compressive yield strength–fracture strain combinations of several Al alloy matrix composites reinforced with different phases (all composites were obtained via the powder metallurgy route).

Matrix Alloy	Nature of the Reinforcement	Reinforcement Composition	Compressive Yield Strength, MPa	Strain at Fracture, %	Reference
Al 5083	Metallic glass-derived crystalline alloy	Al_85_Ni_10_La_5_	729	22.5	[41]
	Ceramic	B_4_C	1058	2.5	[102]
	Ceramic	B_4_C	473	7.2	[103]
Al 6061	Metallic glass	[(Fe_1/2_Co_1/2_)_75_B_20_Si_5_]_96_Nb_4_	570	13	[54]
	Ceramic	MgAl_2_O_4_	370	9	[104]
Al 7075	Metallic glass	Ti_55.5_Cu_18.5_Ni_17.5_Al_8.5_	557	>40	[76]
	Partially crystallized metallic glass	Ti_52_Cu_20_Ni_17_Al_11_	530	27	[67]
	Ceramic	B_4_C	600	20	[105]

**Table 6:** Mechanical properties of selected metallic glass (amorphous alloy)-particle-reinforced MMCs in tension.

Composition of the Material(Matrix + Added/In Situ-Formed Amorphous Reinforcement)	Processing Method	Tensile Yield Strength, MPa	Ultimate Tensile Strength, MPa	Elongation, %	Reference
**Al matrix composites**					
Al + 25 vol.% Ni_60_Nb_40_	Microwave sintering, 823 K; hot extrusion, 623 K	102	120	9.5	[55]
Al-Zn-Ca + 10 vol.% Co_48_Cr_15_Mo_14_C_15_B_6_Tm_2_ clad in layers of Al 5083 alloy	Hot roll bonding, 828 K	245	-	0.5	[73]
Al-4V-2Fe(amorphous phase forms in situ upon solidification)	Rapid solidification of the melt	-	1390	-	[32]
Al 2024 + 40 vol.% Fe_49.9_Co_35.1_Nb_7.7_B_4.5_Si_2.8_	Hot pressing, 673 K, 10 min, 700 MPa; hot extrusion	229	363	4.7	[57]
Al 2024 + 10 vol.% Fe_49.9_Co_35.1_Nb_7.7_B_4.5_Si_2.8_	Hot pressing, 673 K, 10 min, 700 MPa; hot extrusion	176	297	7	[57]
Al + 20 vol.% Al_84_Gd_6_Ni_7_Co_3_ (devitrified during hot extrusion)	Hot pressing, 473 K; hot extrusion, 723 K	93	157	5	[56]
Al 7075 + 6 vol.% Ti_55.5_Cu_18.5_Ni_17.5_Al_8.5_	Hot extrusion, 673 K	500	-	-	[76]
Al + 40 vol.% Fe_50_Cr_25_Mo_9_C_13_B_3_	SPS, 823 K, 10 min, 30 MPa; hot rolling, 823 K	-	254	9.3	[75]
Al + 20 vol.% Zr_48_Cu_36_Ag_8_Al_8_	Hot pressing, 673 K, 640 MPa; hot extrusion, 673 K	155	235	14	[69]
**Mg matrix composites**					
Mg + 10 vol.% Ni_50_Ti_50_	Microwave sintering; annealing, 673 K, 1 h; hot extrusion, 673 K	148	178	2	[84]
**Ti alloy matrix composites**					
Ti_60_Zr_16_V_9_Cu_3_Al_3_Be_9_solidified into a two-phase composite (31 vol.% amorphous phase)	Semisolid processing	1166	1189	9.3	[19]
Ti_67_Zr_11_V_10_Cu_5_Al_2_Be_5_solidified into a two-phase composite (20 vol.% amorphous phase)	Semisolid processing	990	1000	8.4	[19]
**Zr-Ti alloy matrix composite**					
Zr_39.6_Ti_33.9_Nb_7.6_Cu_6.4_Be_12.5_solidified into a two-phase composite (33 vol.% amorphous phase)	Semisolid processing	1096	1210	13	[18]

## Data Availability

Not applicable.
